# The Nutritional Paradox of Obesity: Mechanisms and Clinical Implications of Micronutrient Deficiencies

**DOI:** 10.3390/medsci14020160

**Published:** 2026-03-24

**Authors:** Raluca-Elena Alexa, Raluca Ecaterina Haliga, Bianca Codrina Morărașu, Alexandr Ceasovschih, Oana Sîrbu, Andreea Asaftei, Victorița Șorodoc, Laurențiu Șorodoc

**Affiliations:** 1 Grigore T. Popa University of Medicine and Pharmacy Iasi, 700115 Iasi, Romania; raluca-elena.alexa@umfiasi.ro (R.-E.A.); codrina.morarasu@umfiasi.ro (B.C.M.); alexandr.ceasovschih@yahoo.com (A.C.); oana.sirbu@umfiasi.ro (O.S.); andreea.asaftei@umfiasi.ro (A.A.); victorita.sorodoc@umfiasi.ro (V.Ș.); laurentiu.sorodoc@umfiasi.ro (L.Ș.); 2 2nd Internal Medicine Clinic, St. Spiridon Clinical Emergency Hospital, 700111 Iasi, Romania

**Keywords:** obesity, vitamins, minerals, inflammation

## Abstract

**Background**: Obesity is commonly seen as a condition of overnutrition; however, it is paradoxically associated with micronutrient deficiencies. These deficiencies are clinically relevant and may contribute to the progression of obesity-related comorbidities through interconnected pathways, including chronic low-grade inflammation, oxidative stress, gut dysbiosis, and impaired nutrient absorption. **Objectives:** This narrative review aims to summarize current evidence regarding the prevalence, underlying mechanisms, and clinical consequences of micronutrient deficiencies in individuals with obesity, with particular emphasis on their metabolic implications and potential therapeutic strategies. **Results**: Among individuals with obesity, iron, zinc, magnesium, calcium, vitamin D, vitamin B12, and folate are the most frequently reported deficiencies. These deficiencies arise from multiple mechanisms, including poor diet quality, increased metabolic demands, and compromised gastrointestinal absorption. In addition, obesity-related alterations in pharmacokinetics may further interfere with micronutrient distribution and bioavailability. Together, these mechanisms may lead to various clinical outcomes, such as anemia, immune, metabolic, and cardiovascular dysfunctions, along with cognitive impairment. Although several studies suggest that correcting these deficiencies may improve clinical outcomes, findings remain inconsistent, highlighting the complex and multifactorial pathophysiology underlying micronutrient imbalance in obesity. **Conclusions**: Micronutrient deficiencies represent frequently overlooked contributors to metabolic dysregulation in obesity. Their identification and correction should be considered a central part of the obesity management strategy. A personalized supplementation approach, based on clinical, biological, and pathophysiological characteristics, may provide a complementary support for weight-management treatments.

## 1. Introduction

Obesity is a major public health issue with increasing prevalence worldwide. It represent represents more than a simple excess of adipose tissue [1]. Rather, obesity is a complex disorder characterized by excessive fat accumulation and chronic low-grade inflammation. This condition creates a unique nutritional paradox, in which excessive energy intake coexists with deficiencies in essential micronutrients. Obesity therefore represents an important nutritional challenge that arises from the interaction between undernutrition, due to a variety of causes of poor absorption, and overnutrition, secondary to excessive caloric intake [2]. According to the World Health Organization, the form of malnutrition encountered in obesity is an independent risk factor for the development and progression of various diseases, including dyslipidemias, type 2 diabetes mellitus (T2DM), cardiovascular diseases (CVD), metabolic syndrome (MetS), and certain cancers.

Paradoxically, despite its association with overnutrition, obesity is frequently accompanied by marked deficiencies in several essential micronutrients. These include minerals, such as zinc, iron, calcium, and magnesium, as well as both fat-soluble and water-soluble vitamins [1].

This apparent contradiction can be explained by multiple mechanisms. Poor dietary quality, increased nutritional demands, altered pharmacokinetics, impaired micronutrient absorption, and obesity-associated metabolic and hormonal dysregulation all contribute to this imbalance [2]. These deficiencies may further exacerbate metabolic dysfunction and accelerate the progression of obesity-related comorbidities.

Understanding the bidirectional relationship between micronutrient imbalances and obesity is therefore essential for improving clinical management strategies and optimizing long-term health outcomes.

This review aims to synthesize current evidence on the prevalence, underlying mechanisms, and clinical consequences of major micronutrient deficiencies in individuals with obesity, providing a basis for personalized nutritional interventions.

## 2. Methods of Bibliographic Research

This narrative review was conducted through a structured bibliographic search designed to identify relevant studies addressing micronutrient deficiencies in individuals with obesity, with particular attention to the non-bariatric population. Electronic databases, including PubMed, Google Scholar, Scopus, and Web of Science, were searched for articles published from 2000 to 2026.

The research strategy combined Medical Subject Headings terms and keywords such as “obesity”, “iron deficiency”, “zinc deficiency”, “magnesium deficiency”, “calcium deficiency”, “vitamin D deficiency”, “vitamin B12 deficiency”, and “folate deficiency”. Boolean operators were used to refine the search and improve relevance.

Eligible studies included systematic reviews, meta-analyses, randomized controlled trials, cohort studies, and clinically relevant observational studies that evaluated the prevalence, mechanisms, clinical implications, or management of micronutrient deficiencies in obese populations.

Studies were excluded if they were not available in English, were conducted exclusively on pediatric populations, lacked a clear methodological description, or addressed micronutrient deficiencies unrelated to obesity.

This article is a narrative review rather than a systematic review, so a risk of bias assessment was not performed. However, in order to minimize potential bias, we selected the articles by prioritizing high-quality evidence, such as systematic reviews, meta-analyses, randomized controlled trials, and large observational studies. When available, findings from multiple studies were compared in order to identify trends and areas of discrepancy. Particular attention was given to studies involving adult populations with obesity without prior bariatric surgery in order to reduce heterogeneity related to surgical malabsorption.

## 3. Mechanisms Underlying Nutritional Deficiencies in Obesity

**Figure 1:**
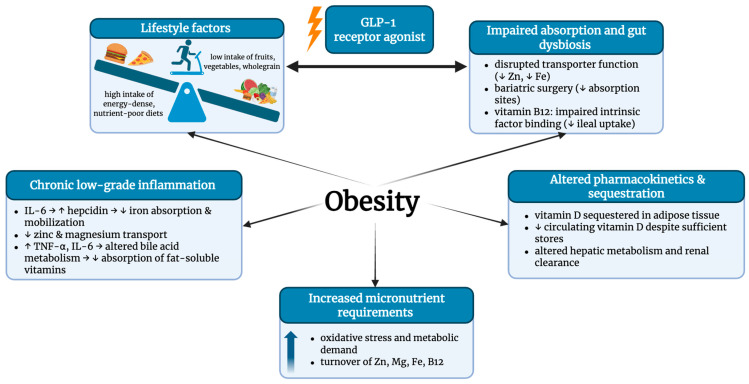
Micronutrients’ deficiencies in obesity—mechanisms and common pathways. Legend: B12—vitamin B12; Fe—iron; GLP-1—glucagon-like peptide-1; IL-6—interleukin-6; Mg—magnesium; TNF-α—tumor necrosis factor-α; Zn—zinc. Created in BioRender. ALEXA, R. (2026) https://BioRender.com/l55b4yp.

Micronutrient deficiencies in patients with obesity result from a complex interplay of inadequate dietary intake, increased metabolic demands, altered nutrient distribution and metabolism, and impaired gastrointestinal absorption. These deficiencies create a background of increased susceptibility, in which each mechanism contributes to distinct yet overlapping pathways, ultimately resulting in a complex state of nutrient depletion and impaired bioavailability (Figure 1).

Micronutrient deficiencies are frequently observed in individuals with obesity, primarily due to poor dietary habits characterized by high consumption of energy-dense foods that are low in fiber, protein, essential micronutrients, and phytochemicals. Within this dietary pattern, ultra-processed foods (UPFs) play a significant role in promoting excessive energy intake while providing limited nutritional value [3]. This pattern is often accompanied by a reduced intake of nutrient-rich foods such as fruits and vegetables, which are primary sources of vitamins and minerals [2]. The adverse health effects of UPFs, including their association with chronic diseases such as T2DM, are well-documented and contribute to metabolic dysfunction [4]. However, nutritional inadequacy alone does not fully account for the observed micronutrient imbalances. Very-low-calorie diets, even when fortified, have also been associated with persistent micronutrient deficiencies, suggesting the involvement of additional factors beyond dietary intake alone [5].

One such factor is the increased micronutrient requirement driven by the metabolic and pathophysiological alterations characteristic of obesity [6]. These alterations include oxidative stress and chronic systemic inflammation, both of which accelerate nutrient depletion [7].

In addition to dietary and metabolic factors, lifestyle interventions such as physical exercise may also influence micronutrient metabolism in individuals with obesity. Physical exercise represents a key component of obesity management and may affect micronutrient homeostasis through several mechanisms. Regular physical activity improves insulin sensitivity, reduces systemic inflammation, and enhances mitochondrial function. Exercise has also been shown to decrease circulating inflammatory markers, which are known to influence micronutrient regulation [2,7].

Elevated requirements for trace elements such as zinc, magnesium, chromium, and manganese have been reported, given their critical roles in carbohydrate and lipid metabolism [8]. Moreover, obesity-associated changes in adiposity, blood composition, renal clearance, and hepatic metabolism may further influence the absorption, distribution, and elimination of micronutrients [2,9].

Altered pharmacokinetics also contribute significantly to micronutrient deficiencies in individuals with obesity. Increased adiposity can disrupt nutrient distribution, metabolism, and excretion through mechanisms involving impaired renal clearance, altered hepatic metabolism, and changes in protein binding and volume of distribution [9]. Physiological alterations such as expanded blood volume, increased cardiac output and organ size, and reduced lean mass can affect the bioavailability of micronutrients [10]. Lipophilic vitamins, particularly vitamins A and D, along with certain minerals (magnesium, selenium, zinc, chromium, and iron), may be sequestered in adipose tissue, leading to reduced circulating levels and metabolic availability [11,12]. The extent to which these mechanisms affect micronutrient status varies between individuals, contributing to the complexity of maintaining nutrient homeostasis in obesity [2,9].

Additionally, gastrointestinal dysfunction may further exacerbate micronutrient malabsorption. Dysbiosis of the gut microbiota, which is common in individuals with obesity, can alter nutrient metabolism and reduce bioavailability [13]. Bariatric surgery, frequently employed to treat morbid obesity, significantly increases the risk of micronutrient deficiencies by reducing both nutrient intake and absorption capacity [14]. The extent of these deficiencies largely depends on the regions of the gastrointestinal tract affected. For instance, the duodenum and jejunum are primary absorption sites for iron, zinc, manganese, selenium, chromium, calcium, and a wide array of vitamins, including A, E, K, folate, and various B-complex vitamins [1]. In contrast, the ileum is responsible for the absorption of vitamin D, vitamin C, and vitamin B12 (following its binding to intrinsic factor in the stomach). Surgical procedures such as gastric bypass disrupt these critical absorption sites, thereby increasing the risk of multiple micronutrient deficiencies [14].

Chronic low-grade inflammation, a hallmark of obesity, also plays a central role in disrupting gastrointestinal function and nutrient absorption. Increased intestinal permeability and persistent gut dysbiosis may reduce nutrient bioavailability [13]. Inflammatory cytokines, notably tumor necrosis factor-alpha (TNF-α) and interleukin-6 (IL-6), can inhibit the expression of intestinal transporters, thereby reducing the absorption of essential minerals like iron, zinc, calcium, and magnesium [2]. Additionally, inflammation may interfere with bile acid metabolism, impairing the absorption of fat-soluble vitamins A, D, E, and K [15]. Gastric and pancreatic dysfunctions may further contribute to deficiencies in vitamin B12, folate, and calcium [16].

Another emerging contributor to nutrient deficiencies in obesity involves incretin-related mechanisms. Incretin hormones such as glucagon-like peptide-1 (GLP-1) and glucose-dependent insulinotropic polypeptide (GIP) regulate appetite, insulin secretion, and nutrient utilizationGLP-1/GIP receptor agonist, now widely used in obesity management due to their effectiveness in promoting weight loss, reducing cardiovascular risk, and lowering the incidence of MetS, may inadvertently lead to micronutrient depletion. Their appetite-suppressing effects, combined with adherence to low-energy diets, can reduce intake of critical nutrients such as iron, calcium, magnesium, vitamin D, and vitamin B12 [17]. While this mechanism is not pathological in nature, it represents a clinically relevant consideration in the comprehensive management of patients with obesity. Moreover, micronutrient deficiencies may alter hormonal signaling pathways, potentially affecting the incretin system and indirectly contributing to micronutrient deficiencies through appetite suppression and reduced dietary intake.

Together, these findings underscore the multifactorial etiology of micronutrient deficiencies in obesity, encompassing dietary inadequacy, increased metabolic demands, altered pharmacokinetics, impaired gastrointestinal absorption, chronic inflammation, and pharmacotherapy. This complexity highlights the importance of implementing individualized nutritional assessments and targeted interventions, including anti-inflammatory dietary strategies and personalized supplementation, to prevent and address micronutrient imbalances in this population.

## 4. Micronutrient Profiles in Obesity

**Figure 2:**
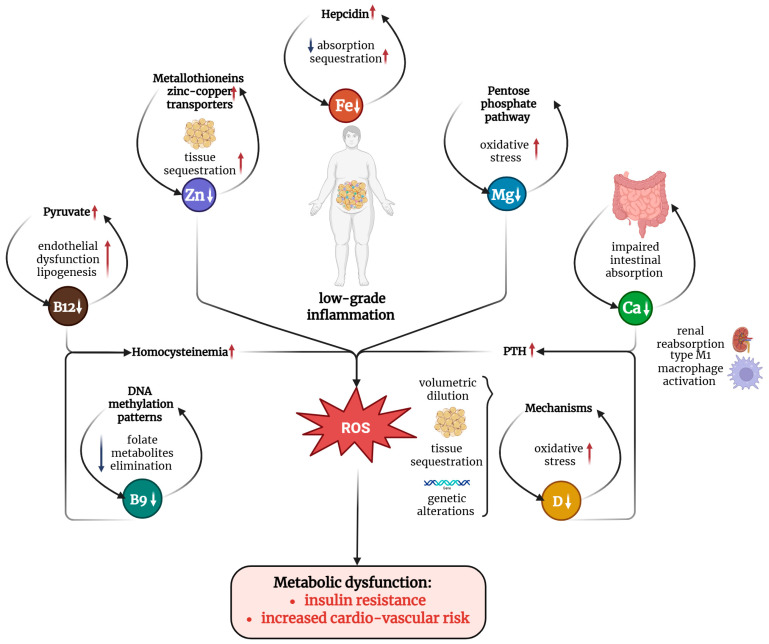
Mechanisms involved in micronutrient deficiencies in obesity: low-grade inflammation contributes to deficiencies in key micronutrients through various mechanisms, which lead to the accumulation of reactive oxygen species and oxidative stress. Collectively, these effects promote insulin resistance and increase cardiovascular risk, hallmark features of metabolic dysfunction associated with obesity. Legend: B9—vitamin B9; B12—vitamin B12; Ca—calcium; D—vitamin D; DNA—deoxyribonucleic acid; Fe—iron; Mg—magnesium; PTH—parathormone; ROS—reactive oxygen species; Zn—zinc. Created in BioRender. ALEXA, R. (2025) https://BioRender.com/dvcokil.

The most prevalent micronutrient deficiencies in obesity include iron, zinc, magnesium, and calcium among minerals, and vitamin D, B12, and folate (B9) among vitamins. Figure 2 illustrates the main mechanisms involved in micronutrient deficiency.

Several studies have attempted to estimate the prevalence of micronutrient deficiencies among individuals with obesity [1,8]. Vitamin D deficiency appears to be the most frequent, affecting approximately 40–80% of individuals with obesity, depending on geographic region and diagnostic thresholds [18,19]. Iron deficiency has been reported in approximately 20–45% of obese women and up to 30% of obese adults overall [20,21,22]. Zinc and magnesium deficiencies have been described in approximately 15–30% of individuals with obesity [23,24,25]. Vitamin B12 and folate deficiencies appear less frequent but remain clinically relevant, particularly in patients with metabolic syndrome or diabetes [26,27].

### 4.1. Iron Deficiency in Obesity

#### 4.1.1. Mechanisms of Deficiency in Obesity

Iron deficiency is one of the most prevalent micronutrient deficiencies in patients with obesity, being closely linked to chronic low-grade inflammation and hormonal dysregulation. The inflammatory environment linked to obesity is mediated by adipose tissue, an active endocrine organ that secretes pro-inflammatory cytokines, including IL-6 and TNF-α. Approximately one-third of circulating IL-6 originates from adipose tissue, contributing to elevated hepcidin levels and disrupted iron homeostasis [20,28].

Hepcidin, a key regulator of iron metabolism, binds to ferroportin and induces the internalization of this transmembrane protein, leading to its degradation and reduced cellular iron transport. These outcomes result in suppressed dietary iron absorption and reduced serum iron levels, along with functional iron deficiency due to impaired mobilization of iron from storage sites such as macrophages, the liver, and the spleen. As a mechanistic chain, chronic inflammation further stimulates hepcidin synthesis, leading to iron sequestration and iron deficiency, despite adequate intake [29].

Due to chronic inflammation, individuals with obesity often present with reduced serum iron levels, elevated soluble transferrin receptor concentrations, and increased physiological iron demands. Moreover, epidemiological studies have demonstrated a strong association between obesity and iron deficiency, particularly in postmenopausal women and adults with higher fat mass [20,21,30]. Additionally, iron supplementation appears less effective in individuals with obesity due to persistently high hepcidin levels, which inhibit absorption [31].

Importantly, weight loss interventions through dietary restriction, exercise, or bariatric surgery have been shown to improve iron homeostasis by reducing inflammation, hepcidin levels, and improving iron absorption [32,33]. In this context, it has been suggested that a reduction in body mass index (BMI) may be associated with decreased hepcidin and leptin levels, resulting in increased transferrin saturation and improved iron status [34]. Structured exercise programs have been shown to decrease C-reactive protein (CRP), IL-6, ferritin, and hepcidin, while bariatric surgery significantly reduces IL-6 and hepcidin, enhancing iron absorption [35,36].

#### 4.1.2. Clinical Implications

Iron deficiency in obesity exerts widespread effects on overall health. In individuals with obesity, disrupted iron metabolism further contributes to gut dysbiosis, neurodegenerative processes, oxidative stress, and hepatic lipid accumulation [37].

Reduced hemoglobin levels contribute to anemia, which in turn leads to symptoms such as fatigue, weakness, diminished physical endurance, and impaired metabolic function [38]. Cognitive function may also be affected, as iron deficiency disrupts neurotransmitter synthesis and oxygen transport, leading to memory deficits and slower cognitive processing. Additionally, inadequate iron levels can exacerbate chronic inflammation and oxidative stress, worsening obesity-related metabolic disturbances [22,37]. Impaired iron availability also affects mitochondrial function and insulin signaling, increasing the risk of insulin resistance (IR) and T2DM [39]. Moreover, iron deficiency weakens immune function, reducing immune cell activity, increasing susceptibility to infections, and slowing wound healing [40]. Cardiovascular risks are also heightened, as iron deficiency is linked to endothelial dysfunction and hypertension [41].

### 4.2. Zinc Deficiency in Obesity

#### 4.2.1. Mechanisms of Deficiency in Obesity

Zinc is a vital trace element involved in numerous physiological reactions, including lipid and glucose metabolism, antioxidant defense, and the modulation of inflammatory pathways. In individuals with obesity, serum levels are frequently reduced [23,24].

Several mechanisms have been proposed to explain zinc deficiency. Firstly, chronic low-grade inflammation associated with obesity leads to increased expression of metallothioneins and zinc-copper transporters, which sequester zinc in hepatocytes and adipocytes, thereby reducing its bioavailability. Secondly, dietary factors also play a role, as individuals with obesity often consume diets low in zinc-rich foods [42].

Zinc is an essential component of zinc-alpha-2-glycoprotein, which is involved in the regulation of leptin levels. Obesity is typically associated with elevated leptin levels and leptin resistance. The dysregulation of leptin signaling impairs the interaction between the hormone and its receptor, prompting adipose tissue to secrete more leptin, thereby perpetuating obesity and the associated inflammatory state. Furthermore, leptin promotes the secretion of pro-inflammatory cytokines, such as IL-6 and TNF-α, which further contribute to systemic chronic inflammation and zinc depletion [42,43].

Another key mechanism contributing to zinc deficiency in obesity is oxidative stress, which results from an imbalance between free radicals and antioxidants [44]. Zinc is a cofactor for several antioxidant enzymes, including superoxide dismutase. When zinc levels are insufficient, the antioxidant defense system is compromised, leading to increased lipid peroxidation and production of reactive oxygen species (ROS), which are central to the pathogenesis of obesity-related metabolic dysfunctions. These oxidative and inflammatory pathways interact, creating a vicious cycle that perpetuates zinc deficiency and metabolic deterioration [24,45].

#### 4.2.2. Clinical Implications

Zinc deficiency in individuals with obesity has significant clinical consequences and may manifest through symptoms such as delayed wound healing, hair loss, skin lesions, impaired taste, infertility, and anemia. Notably, in bariatric surgery patients, the prevalence of zinc deficiency is high both preoperatively and postoperatively, particularly in those undergoing Roux-en-Y gastric bypass or mini-gastric bypass [46,47]. Regarding zinc’s implication in metabolic syndrome, there are contradictory correlations between serum zinc levels and anthropometric measurements or lipid profile, suggesting a potential link between zinc status and cardiovascular risk profile [24,44,45].

Beyond that, zinc may act as an insulin mimetic, its deficiency being associated with insulin resistance and impaired pancreatic β-cell function, and thereby contributing to glycemic dysregulation in obesity and MetS [42].

### 4.3. Magnesium Deficiency in Obesity

#### 4.3.1. Mechanisms of Deficiency in Obesity

Obesity, MetS, and T2DM are interconnected through shared pathophysiological mechanisms driven by chronic low-grade inflammation, in which magnesium deficiency plays a pivotal role. This deficiency is highly prevalent among individuals with these conditions and has been consistently associated with an increased risk of T2DM, not only due to its direct metabolic effects but also through its contribution to systemic inflammation, both directly and via alterations in the gut microbiota [25,48].

Poor dietary patterns commonly observed in individuals with obesity—characterized by high energy density and low micronutrient content—frequently result in inadequate magnesium intake. Several studies have reported an inverse correlation between dietary magnesium and anthropometric measurements, such as BMI and waist circumference [25,49,50]. Moreover, magnesium supplementation has been shown to reduce adipose tissue accumulation and levels of inflammatory biomarkers, underscoring its potential role in the management of obesity-related complications [51].

In individuals with obesity, excessive intake of refined carbohydrates increases hepatic glucose metabolism, which depends on magnesium-dependent enzymatic pathways. Magnesium is essential for activating vitamin B1 (thiamine) to thiamine diphosphate (TDP), a coenzyme critical to oxidative glucose metabolism. Low intracellular magnesium and TDP levels reduce pyruvate dehydrogenase activity, shifting metabolism toward the pentose phosphate pathway. This shift leads to excessive NADPH production, which promotes lipogenesis and contributes to increased adiposity, dyslipidemia, IR, and T2DM [52,53,54,55].

Chronic subclinical magnesium deficiency contributes to inflammatory responses through cytokine secretion and oxidative stress, further exacerbating pre-existing inflammation [48]. Additionally, magnesium deficiency has been recognized as a modifiable risk factor for hypertension, CVD, and MetS [56,57].

Moreover, vitamin D deficiency is a common feature in obese individuals regardless of diabetic status. The coexistence of magnesium and vitamin D deficiencies is associated with a higher risk of IR, chronic kidney disease progression, MetS, and cardiovascular complications [18,19]. Magnesium is a vital cofactor for both vitamin D synthesis and activation. Optimal magnesium status can enhance vitamin D bioavailability and its cardiometabolic effects. Interventional studies have shown that vitamin D supplementation can increase serum magnesium levels, possibly through enhanced renal retention, underscoring the interdependence of these micronutrients [58,59,60].

#### 4.3.2. Clinical Implications

Regarding magnesium deficiency symptoms in patients with increased adiposity, no significant differences in clinical signs and symptoms have been reported compared with non-obese individuals. Magnesium deficiency’s main characteristic is its involvement in metabolic dysregulation, systemic inflammation, insulin resistance, and cardiovascular pathology, being a modifiable risk factor. Its correction through dietary strategies and supplementation may improve glucose metabolism, enhance insulin sensitivity, and reduce cardiometabolic risk. Due to its wide range of biological functions, magnesium repletion should be considered an important part of obesity management strategies, especially in patients with associated metabolic dysfunctions [25].

### 4.4. Calcium Deficiency in Obesity

#### 4.4.1. Mechanisms of Deficiency in Obesity

Calcium is an essential mineral involved in bone metabolism, muscle contraction, nerve transmission, vascular function, and intracellular signaling. In individuals with obesity, calcium homeostasis is frequently disrupted, leading to a higher risk of hypocalcemia and long-term metabolic complications [61].

Several interrelated mechanisms contribute to calcium deficiency in individuals with obesity. A key contributor is vitamin D deficiency, which is highly prevalent in obese populations. As a fat-soluble vitamin, vitamin D can be sequestered in adipose tissue due to its fat-soluble nature, resulting in lower bioavailability and impaired intestinal calcium absorption. Additional mechanisms, such as volumetric dilution, may also contribute to reduced circulating vitamin D levels in individuals with obesity. Moreover, vitamin D deficiency impairs calcium uptake, even in the presence of normal dietary intake [62,63,64,65,66,67].

Additionally, chronic low-grade inflammation, common in obesity, negatively affects calcium metabolism. Pro-inflammatory cytokines, such as TNF-α and IL-6, interfere with bone remodeling by enhancing osteoclast activity and reducing osteoblast function. This imbalance promotes bone resorption and increases calcium mobilization from the skeletal system [61].

Another mechanism involves IR, a hallmark of obesity. Insulin facilitates renal calcium reabsorption and modulates parathyroid hormone (PTH) secretion [68]. In IR states, calcium reabsorption is reduced, and PTH levels are increased, which may exacerbate calcium imbalance [69,70].

Moreover, dietary habits associated with obesity often contribute to low calcium intake. High consumption of processed foods, soft drinks rich in phosphates, and low intake of dairy products can disturb the calcium–phosphorus ratio, leading to impaired calcium absorption.

#### 4.4.2. Clinical Implications

One of the most well-recognized consequences is an increased risk of osteopenia and osteoporosis, resulting from impaired bone mineralization and accelerated bone turnover [71]. This risk is further exacerbated in individuals with obesity who undergo bariatric surgery, particularly procedures that induce malabsorption [14].

Calcium deficiency also plays a role in the pathophysiology of hypertension. Low serum calcium may stimulate the renin–angiotensin–aldosterone system, increase vascular smooth muscle tone, and promote arterial stiffness. These pathways contribute to elevated blood pressure and increased cardiovascular risk in obese patients [72].

In addition, insufficient calcium intake and low serum calcium levels have been associated with abnormal lipid profiles, IR, and impaired glucose metabolism—all key components of metabolic syndrome [70]. Furthermore, alterations in adipocyte metabolism and intracellular calcium signaling pathways may influence lipid storage and lipolysis, suggesting a potential link between calcium status and obesity [69].

### 4.5. Vitamin D Deficiency in Obesity

#### 4.5.1. Mechanisms of Deficiency in Obesity

Vitamin D is a fat-soluble vitamin, and adipose tissue serves as a major site for its storage and utilization. However, the mechanisms involved in vitamin D storage in adipose tissue are not fully understood. Possible explanations include sequestration in adipose tissue, volumetric dilution, impaired metabolism, and genetic mechanisms [73].

Although vitamin D levels are decreased in individuals with obesity, their distribution pattern remains consistent across all BMI ranges, suggesting vitamin D sequestration in adipose tissue [74]. Moreover, vitamin D deficiency may contribute to lipogenesis through PTH by increasing calcium influx in adipocytes and decreasing lipoprotein lipase levels. In addition, secondary hyperparathyroidism associated with vitamin D deficiency may increase adipose tissue mass, suggesting that gender and PTH levels contribute to obesity’s enhancement [75].

Volumetric dilution has been proposed as a potential mechanism underlying vitamin D deficiency in obesity, as body weight-adjusted vitamin D levels show no significant differences between obese and lean individuals [73]. Notably, individuals with obesity have been shown to have higher concentrations of vitamin D stores, with omental adipose tissue containing greater levels than subcutaneous fat, as demonstrated in tissue analyses [74].

Elevated serum levels of proinflammatory cytokines—such as IL-6, IL-8, and TNF-α—observed in individuals with obesity suggest that chronic low-grade inflammation may originate from preadipocytes or result from apoptosis of hypertrophic adipocytes. In addition, adipose tissue inflammation promotes the recruitment and polarization of macrophages toward the proinflammatory M1 phenotype [73].

Vitamin D deficiency influences immune mechanisms by activating pathways that contribute to obesity-related chronic inflammation and immune dysregulation. Given its immunomodulatory properties, vitamin D exerts its effects through the vitamin D receptor (VDR) and by activating nuclear signaling pathways [73,76].

Vitamin D deficiency contributes to metabolic dysregulation by promoting IR, primarily through reduced expression of glucose transporters. Additionally, disruption of the antioxidant defense system and increased oxidative stress further impair cellular metabolism, ultimately leading to apoptosis [77].

Another mechanism involves genetic alterations, most notably those related to the VDR, which is often overexpressed in individuals with obesity. The underlying mechanisms are more complex, since VDR plays an important role in the transcription of genes involved in cell proliferation, cell differentiation, and angiogenesis [78], along with an increased activity of peroxisome proliferator-activated receptor γ, fatty acid binding protein, and lipoprotein lipase [73].

#### 4.5.2. Clinical Implications

Vitamin D deficiency in obesity leads to MetS, numerous studies demonstrating its implication in T2DM and cardiovascular risk [79,80,81]. Also, its implication in bone metabolism during menopause is well known, osteopenia and increased body mass contributing to cardiometabolic risk, along with increased levels of PTH in postmenopausal women [82].

Vitamin D deficiency activates the renin–angiotensin–aldosterone system, thereby contributing to elevated blood pressure. In addition, vitamin D exerts direct vasoprotective effects by enhancing endothelial function and reducing vascular inflammation; consequently, its deficiency may lead to endothelial dysfunction [79,83]. The role of vitamin D in atherosclerosis is further supported by its association with an atherogenic lipid profile, chronic inflammation, endothelial dysfunction, and hypertension [84].

### 4.6. Vitamin B12 Deficiency in Obesity

#### 4.6.1. Mechanisms of Deficiency in Obesity

Although vitamin B12 deficiency has multiple causes, including inadequate intake, genetic predisposition, and malabsorption due to bariatric surgery or intestinal disorders, in individuals with obesity, it is most commonly attributed to poor dietary intake or the use of medications such as metformin and proton pump inhibitors [26].

Vitamin B12 deficiency plays a key role in the pathogenesis of obesity and MetS due to its association with chronic inflammation and IR. Low levels of vitamin B12, along with a functional deficiency characterized by increased binding of vitamin B12 to transcobalamin I and III, and a decreased binding to transcobalamin II, contribute to hyperhomocysteinemia. Increased levels of homocysteine lead to oxidative stress, inflammation, and endothelial dysfunction, along with lipogenesis by inhibiting fatty acid oxidation [27,85].

Moreover, available evidence from preclinical and clinical studies suggests that low serum levels of vitamin B12 can promote hepatic lipid accumulation by activating lipogenic pathways or impairing fatty-acid oxidation through methylmalonic acid-related pathways [26,86].

Medical conditions such as polycystic ovary syndrome (PCOS) and vitamin B12 deficiency may contribute to IR and obesity through disruptions in B12 metabolism and elevated homocysteine levels. These alterations impair methylation processes, reduce lean tissue deposition, and promote adiposity [87].

Patients undergoing gastric surgery are at an increased risk of micronutrient malabsorption, including vitamin B12 deficiency, which may be compounded by a pre-existing deficiency associated with obesity [14].

Moreover, the chronic use of metformin in patients with diabetes or MetS has been associated with an increased risk of vitamin B12 deficiency, likely due to impaired calcium-dependent absorption of the B12-intrinsic factor complex in the ileum. This association appears to be independent of BMI and is linked to elevated homocysteine levels, anemia, and peripheral neuropathy [88].

These alterations may also contribute to neuropsychiatric dysfunction, as vitamin B12 deficiency is more frequently observed in elderly patients and in individuals with psychiatric disorders [89].

#### 4.6.2. Clinical Implications

To date, no studies have specifically investigated differences in the clinical manifestations of vitamin B12 deficiency between obese and non-obese individuals. B12 deficiency may manifest through a multitude of signs and symptoms, independent of BMI. The most frequently reported symptoms include severe fatigue, reduced energy levels, and sensations of dizziness or light-headedness, all of which can significantly impair daily functioning and overall quality of life [90].

The most common hematological sign is megaloblastic anemia, and, in severe cases, pancytopenia may occur [91].

Neurological symptoms of vitamin B12 deficiency may include peripheral neuropathy, typically presenting as tingling and numbness in the extremities. In more severe cases, spinal cord degeneration may occur, resulting in gait disturbances, impaired balance, and limb weakness. These manifestations may be further exacerbated by physical inactivity, a common feature in individuals with obesity [90,91].

Some patients may experience glossitis, anorexia, and constipation or diarrhea, which can be mild or overlooked, especially in obese patients [91].

In addition to physical symptoms, neuropsychiatric manifestations, such as depression, delirium, mania, mood disorders, and cognitive decline, may also occur in obese individuals [91].

In young females, maternal vitamin B12 deficiency is often associated with increased adiposity, particularly during the first trimester of pregnancy. Furthermore, a study conducted by Sukumar et al. [85] demonstrated that vitamin B12 insufficiency is linked to a higher risk of developing gestational diabetes mellitus compared to individuals with normal B12 levels. The same study also found that vitamin B12 deficiency during pregnancy increases the risk of macrosomia, independently of gestational diabetes status [85].

### 4.7. Folate Deficiency in Obesity

#### 4.7.1. Mechanisms of Deficiency in Obesity

Folate deficiency in obese patients is linked to both lower intake and reduced serum levels. Several studies have shown that these mechanisms may occur independently of overall diet quality, urinary excretion, dilution effects, or increased utilization [92].

Moreover, folate deficiency in obesity may contribute to epigenetic dysregulation by impairing one-carbon metabolism, resulting in altered DNA methylation patterns. These changes affect the expression of genes involved in metabolic pathways, fat storage, and disease susceptibility. Additionally, folate deficiency may influence key regulatory genes such as DNMTs, RXRα, and IGF2/H19, which play central roles in methylation processes, thereby promoting adverse metabolic traits and increasing the long-term risk of obesity and related disorders [93].

Although folate is not a direct substrate of cytochrome P450 2E1, enhanced CYP2E1 activity increases intracellular oxidative stress, which may promote oxidative degradation of reduced folate species and increase folate turnover, thereby contributing to relative folate deficiency despite adequate intake. Additionally, in obese patients, the impaired excretory capacity decreases the elimination of folate metabolites, leading to accumulation of oxidation products rather than usable folate [94,95,96,97,98,99].

#### 4.7.2. Clinical Implications

Folate deficiency may also impair insulin sensitivity and lipid metabolism. Several studies have reported that elevated homocysteine levels—directly associated with MetS and increased cardiovascular risk—are inversely correlated with folate concentrations. While inflammation and oxidative stress are well-established features of obesity, elevated homocysteine levels are believed to further contribute to this pathogenic milieu [100].

Obesity in women of childbearing age has been associated with altered folate pharmacokinetics, characterized by lower serum folate concentrations, reduced peak serum responses following oral folate administration, and elevated red blood cell folate levels. These findings suggest a redistribution of folate from the circulation into peripheral tissues, which may lead to reduced folate availability during early embryogenesis and contribute to the increased risk of neural tube defects. Moreover, these findings support the need for revised folate intake recommendations based on BMI [101].

## 5. Micronutrient Deficiency and Visceral Complications of Obesity: Implications for Metabolic Dysfunction-Associated Steatotic Liver Disease and Chronic Kidney Disease

Visceral complications contribute substantially to obesity-related morbidity. Metabolic dysfunction-associated steatotic liver disease (MASLD) and chronic kidney disease (CKD) represent two highly prevalent and frequently overlapping conditions. Contemporary clinical guidelines underscore their shared pathophysiological background, namely insulin resistance, chronic low-grade inflammation, oxidative stress, lipotoxicity, and endothelial dysfunction, within which micronutrient deficiencies may operate as amplifiers of tissue injury and disease progression, even when not primary etiological drivers [102,103].

In MASLD, disturbances in micronutrients involved in redox regulation and immunometabolic control appear particularly relevant. Vitamin D deficiency, commonly observed in individuals with obesity, has been associated with both the presence and severity of hepatic steatosis, and in some studies, with advanced fibrosis. Vitamin D signaling modulates inflammatory pathways, macrophage activation, and insulin sensitivity, central mechanisms to the transition from simple steatosis to steatohepatitis and fibrogenesis [104,105]. Moreover, impaired antioxidant defense contributes to hepatocellular vulnerability. Vitamin E, a key lipid-soluble antioxidant, has demonstrated therapeutic benefit in selected patients with steatohepatitis, reinforcing the pathogenic role of oxidative stress in metabolic liver injury [106].

Trace elements, including zinc, selenium, copper, and iron, are essential cofactors for mitochondrial enzymes and antioxidant systems, such as superoxide dismutase and glutathione peroxidase. Emerging evidence in metabolic fatty liver disease suggests that deficiencies or dysregulation of these micronutrients may exacerbate lipid peroxidation, endoplasmic reticulum stress, mitochondrial dysfunction, and inflammatory signaling cascades, thereby facilitating progression toward steatohepatitis and fibrosis [107,108]. Notably, in obesity, micronutrient abnormalities are frequently functional rather than absolute, reflecting altered transport proteins, inflammatory redistribution, and reduced circulating bioavailability despite preserved or increased tissue stores.

In parallel, CKD associated with obesity is influenced by micronutrient status through mechanisms involving persistent inflammation, vascular dysfunction, oxidative stress, and mineral metabolism disturbances. Patients with CKD are predisposed to micronutrient deficiencies due to dietary restrictions, altered gastrointestinal absorption, anorexia, and impaired renal handling [109]. Vitamin D deficiency assumes particular relevance in CKD, as reduced renal 1α-hydroxylase activity limits calcitriol synthesis, contributing to CKD–mineral and bone disorder. KDIGO guidelines recommend assessment of 25(OH)D concentrations and correction of deficiency or insufficiency according to general population strategies, adapted to CKD-specific mineral metabolism abnormalities [110].

Disturbances in trace elements, particularly zinc, selenium, copper, and iron, have been linked to impaired antioxidant defenses, altered erythropoiesis, immune dysfunction, and heightened cardiovascular risk in CKD populations [109]. A recent systematic review evaluating zinc supplementation in adults with CKD suggests potential improvements in selected biochemical and clinical outcomes, supporting the biological plausibility of zinc status as a modifiable factor in this context [111]. Furthermore, accumulating data associate low magnesium levels with endothelial dysfunction and accelerated vascular calcification in CKD, mechanisms closely linked to cardiovascular mortality and possibly renal progression [112].

Taken together, the interplay between micronutrient deficiencies and visceral complications in obesity appears bidirectional. Deficiencies may heighten susceptibility to hepatic and renal oxidative injury, while progressive organ dysfunction further disrupts micronutrient metabolism and distribution. These considerations justify targeted micronutrient evaluation in obese individuals with established MASLD, particularly those with suspected steatohepatitis or fibrosis, and in patients demonstrating declining renal function. However, current interventional evidence does not support indiscriminate supplementation, and therapeutic decisions should remain individualized and guided by documented deficiencies and clinical context [102,109].

## 6. Supplementation Approaches to Micronutrient Deficiencies in Obesity

Micronutrient supplementation in individuals with obesity has yielded inconsistent results. These discrepancies may be attributed to unstandardized study designs, heterogeneous populations, and variations in supplementation regimens. Table 1 summarizes several supplementation regimens reported in the literature, highlighting the diverse outcomes observed in patients who have not undergone metabolic bariatric surgery.

Micronutrient deficiencies in the context of metabolic bariatric surgery represent another well-studied area. A meta-analysis conducted by Tang et al. [132] concluded that micronutrient supplementation prior to bariatric surgery is advisable; however, its effectiveness depends on several factors, including the type of deficiency, the administered dose, duration of treatment, and route of administration [132]. Moreover, preoperative nutrient deficiencies may influence postoperative outcomes, reinforcing the recommendation to assess and correct specific deficiencies both before and after bariatric surgery [133].

In obese patients with iron deficiency, oral supplementation is restricted due to increased hepcidin levels and persistent inflammation [31]. However, intravenous iron administration is more effective, but diet-induced weight loss can reduce chronic inflammation and lower hepcidin levels, thereby enhancing iron absorption [113]. Hence, excessive iron supplementation may have adverse effects by disrupting gut microbiota composition, thereby contributing to or perpetuating dysbiosis and, consequently, chronic inflammation [134].

Across multiple studies, zinc supplementation has shown no consistent effect on BMI or weight management, with findings remaining contradictory. A meta-analysis concluded that the impact of zinc supplementation may depend on the health status of the individuals, with beneficial effects on BMI reduction observed only at higher doses (100 mg/day) [135]. Hence, zinc may play an important role in cognitive health among obese patients, with or without prior neurological disorders [117].

Regarding targeted treatment, a Cochrane meta-analysis [136] concluded that high-dose calcium supplementation (>1 g/day) may influence body weight. Combined with vitamin D, small doses of calcium could have a positive effect on body weight [121]. Given calcium’s involvement in glucose and lipid metabolism, its contribution to metabolic homeostasis is significant but influenced by multiple factors, which may explain the contradictory findings reported across studies [137].

Most studies on magnesium supplementation have focused on its effects on adiposity-related comorbidities in both obese and non-obese populations. Magnesium supplementation may reduce the risk of MetS and T2DM, as well as decrease waist circumference, although these effects appear to depend on comorbid conditions and administered dosages [138,139].

The result of vitamin D supplementation on obese patients is inconsistent regarding its effect on weight loss or other metabolic effects. Most of the studies suggest that moderate doses of vitamin D (2000 UI/day) correct its deficit, but the results depend on BMI and baseline vitamin D level [125], and higher doses (above 4000 UI/day) were linked to better blood pressure and metabolic profile [140]. However, inconsistencies in LDL cholesterol serum levels observed during weight loss may be attributed to reduced calcium levels, suggesting that calcium co-supplementation could help mitigate this effect [141]. Moreover, after correction of vitamin D deficiency, maintenance doses should be administered [126]. Some researchers support the preventive use of vitamin D to improve adipose tissue function, particularly in individuals with morbid obesity; however, current evidence remains insufficient to support this recommendation.

Regarding vitamin B12 supplementation, most available studies have focused on patients who have undergone bariatric surgery. To date, no studies have specifically investigated vitamin B12 deficiency in individuals with obesity who have not undergone such procedures. Moreover, current treatment protocols for vitamin B12 deficiency are applied uniformly, regardless of BMI.

Findings from studies on folate supplementation in individuals with obesity remain inconsistent. A meta-analysis conducted by Jafari et al. [142] concluded that folate supplementation does not significantly affect BMI or body weight in the general population; however, it was associated with a reduction in BMI among patients with hyperhomocysteinemia and in women with PCOS, likely due to improvements in IR and decreased leptin levels. These results support the potential benefit of folate supplementation in individuals with elevated homocysteine levels or in women with PCOS [142]. Moreover, folate administration decreases the cardiovascular risk by lowering homocysteine levels, particularly in overweight individuals [143].

These findings highlight the need for further research on personalized micronutrient interventions. Future randomized controlled trials are required to clarify the potential benefits of targeted supplementation strategies for improving metabolic profile [100,130].

## 7. Limitations of the Current Literature and Future Research Trajectories

Despite the growing interest in micronutrient deficiencies in obesity, several methodological and conceptual limitations restrict the strength of current conclusions and their translation into clinical practice.

Firstly, most available data derive from cross-sectional or observational studies, limiting causal inference. Reverse causality remains a significant concern, as the altered pharmacokinetics, chronic inflammation, and organ dysfunction characteristic of obesity may modify circulating micronutrient levels, complicating the distinction between primary deficiency and secondary redistribution [107,110]. Moreover, heterogeneity across studies, including variability in BMI categories, comorbidities, age, adiposity phenotypes, sex, ethnicity, and dietary patterns, impairs generalizability and comparability [103,107].

Secondly, assessment of micronutrient status is frequently based solely on serum concentrations, which may not accurately reflect intracellular stores or functional bioavailability, particularly in the context of inflammation-driven shifts in transport proteins and tissue sequestration [107,110]. Standardized diagnostic cut-offs specific to individuals with obesity are lacking, and functional biomarkers are seldom incorporated.

Thirdly, randomized controlled trials investigating supplementation strategies show considerable heterogeneity in study design, duration, baseline deficiency status, dosing regimens, and outcome measures [132,142]. Many trials are short-term and primarily evaluate surrogate metabolic markers rather than clinically meaningful endpoints, such as progression of MASLD, renal decline, or cardiovascular events. Additionally, nutrient–nutrient interactions and combined deficiencies are rarely addressed.

Future research should focus on well-designed longitudinal studies to clarify temporal and bidirectional relationships between micronutrient imbalances and obesity-related complications. Standardization of deficiency definitions and incorporation of functional biomarkers are warranted. Large, adequately powered trials targeting clearly defined phenotypes, including patients with MASLD, CKD, or post-bariatric surgery, are necessary to determine whether tailored supplementation improves clinically relevant outcomes [132,144,145].

An important clinical consideration is the distinction between micronutrient deficiencies directly related to obesity-related pathophysiology and those secondary to comorbid conditions such as medical treatments. In many cases, micronutrient deficiencies observed in obese populations may be influenced by factors such as diabetes-related pharmacotherapy (e.g., metformin), proton pump inhibitors, chronic kidney disease, or bariatric surgery. These factors may independently influence micronutrient metabolism, absorption, and bioavailability. Consequently, the interpretation of micronutrient status in obesity requires careful consideration of these potential confounding factors [9,14,26,109]. This distinction is particularly relevant for clinical practice, as management strategies should address the underlying cause of deficiency rather than assuming that all micronutrient abnormalities are intrinsic to obesity.

## 8. Conclusions

Micronutrient deficiencies in obese patients represent a paradoxical issue; that is, an imperfect balance between hyper-energy status vs. mineral and vitamin micronutrient deficiencies. These profile features contribute to metabolic dysfunction and chronic inflammation promotion. Published data about this topic show that in obesity, deficits are more likely functional than absolute ones. For these scenarios, micronutrient and vitamin status analyses should consist of an extensive and integrative evaluation that includes clinical and biological individual peculiarities.

Targeted interventions should include anti-inflammatory dietary patterns, appropriate micronutrient repletion, and longitudinal monitoring. These strategies are essential for optimizing metabolic health in improving clinical outcomes.

Therefore, further research is needed to investigate the role of deficiencies supplementation in the prevention and management of obesity-related diseases to establish population-specific guidelines for micronutrient screening indications.

## Figures and Tables

**Table 1:** Different regimens used in micronutrient deficiencies in patients with obesity.

Micronutrient	Doses	Results
Iron	5 mg FeSO_4_ ± 31.4 mg ascorbic acid, p.o.	Ascorbic acid has a limited effect on iron absorption in overweight and obese patients due to hepcidin involvement [28].
	200 mL hydroxy sucrose iron/week, 5 weeks, i.v.	Increased ferritin and hemoglobin levels post-treatment in patients with obesity and ferritin < 50 ng/mL [113].
Zinc	100 mg elemental Zn, p.o.	Increased serum leptin and anthropometric measurements were observed in hemodialysis patients [114].
	30 mg Zn gluconate, p.o.	Improved BMI, body weight, and serum TG, without interfering with lipid profile or serum glucose [115].
	30 mg/day Zn sulfate, p.o.	Combined with a restrictive calorie diet, Zn supplementation improved anthropometric measurements, inflammatory and metabolic biomarkers [116].
	30 mg/day chelated Zn, 12 weeks, p.o.	Partially improved cognitive function, without interfering with metabolic markers, except for IL-1β [117].
Magnesium	365 mg/day Mg, 3 months, p.o.	Improved metabolic profile, without significant changes on BMI [118].
	300 mg/day Mg sulfate, 6 months, p.o.	Improved metabolic profile in overweight patients with IR [119].
	500 mg/day Mg, p.o.	Magnesium supplementation improved sleep indices in the elderly, without influence on anthropometric indices [120].
Calcium	600 mg Ca + 125 IU vitamin D3, p.o.	Combined with a low-energy diet, calcium and vitamin D3 augmented weight loss in healthy young adults [121].
	500 mg Ca carbonate + 1000 UI vitamin D3, 12 weeks, p.o.	Combined with vitamin D and an energy-restrictive diet, calcium supplementation in patients with vitamin D deficiency and NAFLD improved glucose and lipid metabolism [122].
	1000 mg/day Ca carbonate 50,000 IU/week vitamin D3 50,000 IU/week vitamin D3 + 1000 mg/day Ca carbonate 8 weeks	Co-administration of vitamin D and calcium improved systemic inflammation in T2DM patients with vitamin D deficiency [123]. Co-administration of vitamin D and calcium improved metabolic profile in women with PCOS and vitamin D deficiency [124].
Vitamin D	600–12,000 IU/day	Moderate doses of vitamin D (1600–2000 UI/day) increased the vitamin D serum concentrations [125].
	1000–4000 IU/day	Higher doses (4000 UI/day) increase C-peptide secretion and therefore decrease IR [126].
	7000 IU/day	Increase vitamin D levels and decrease PTH serum levels, without interfering with adipose tissue [127].
	300–12,185 IU/day	Higher doses (≥4000 UI/day) increased vitamin D serum levels, improved blood pressure, and lipid profile [128].
Folate	1 mg/day or 5 mg/day, p.o.	5 mg/day of folic acid supplementation improved metabolic profile of overweight women with PCOS [129].
	5 mg/day, p.o.	Improved BMI in those with high serum homocysteine levels ≥15 μmol/L at baseline during weight loss program [130]. Improved metabolic profile of patients with T2DM [131].

## Data Availability

No new data were created or analyzed in this study.
